# Human neural organoid modeling of diffuse midline glioma captures the complexity of patient tumors

**DOI:** 10.1007/s11060-026-05515-5

**Published:** 2026-05-07

**Authors:** Jack M. Shireman, Elliot Xie, Connie S. Lebakken, Sudarshawn Damodharan, Kailyn T. Parham, William D. Richards, Rintaro Hashizume, Christina Kendziorski, Mahua Dey

**Affiliations:** 1 Department of Neurosurgery, University of Wisconsin School of Medicine & Public Health, UW Carbone Cancer Center, Madison, WI USA; 2 Department of Biostatistics and Medical Informatics, University of Wisconsin School of Medicine and Public Health, Madison, WI USA; 3 Stem Pharm Incorporated, Madison, WI USA; 4 Department of Pediatrics, Section of Hematology, Oncology & Stem Cell Transplantation, University of Chicago, Chicago, IL USA; 5 Department of Pediatrics, University of Alabama at Birmingham, Birmingham, AL USA; 6 University of Wisconsin School of Medicine & Public Health, 600 Highland Ave, Madison, WI 53792 USA

**Keywords:** Diffuse midline glioma, Planar neural organoids, Animal-free modeling, Neuro-oncology

## Abstract

**Background:**

Diffuse Midline Glioma H3K27-altered (DMG) is an extremely aggressive and lethal childhood brain cancer that grows within the midline structure of the brain. Current treatment options are only palliative, making DMG in desperate need for therapeutic breakthroughs. One of the major challenges limiting the study of DMG is the lack of reliable preclinical models. In-vivo mouse models are expensive and technically challenging and in-vitro cell culture models lack the essential components of tumor microenvironment (TME) needed to recapitulate the complex biology of these tumors. Scalable human planar neural organoids (PNOs) with multi-cellular make-up can serve as a cost effective and reliable model system to capture DMG biology and allow effective species matched drug testing in-vitro.

**Methods:**

Using 3 separate DMG patient derived xenograft (PDX) cell lines, we spatially profiled a novel scalable human iPSC-derived PNO system containing neurons, functional astrocytes and microglia using the NanoString GeoMx spatial transcriptomics system.

**Results:**

We found that all three cell lines interact with and integrate into the human PNOs, demonstrating favorable growth conditions in a complex co-culture. Across spatially resolved regions of interest (ROI’s) tumor cells individually interact with microglia and astrocytes and transcriptomic profiling of these mixed cell ROI’s shows differences in the genetic signatures of both the normal cells (microglia/astrocytes) and the tumor cells. When compared to biopsies obtained directly from DMG patients, DMG cells within PNOs correlate strongly at both transcriptomic and proteomic levels. The multi-cellular PNOs also enabled drug target bystander toxicity screening not possible in a traditional tumor cell only monoculture.

**Conclusion:**

This study provides a proof-of-concept for scalable PNO modeling for DMG and underscores the translational relevance of this model system.

**Supplementary Information:**

The online version contains supplementary material available at 10.1007/s11060-026-05515-5.

## Introduction

Brain tumors develop in an incredibly complex central nervous system (CNS) microenvironment consisting of neurons, astrocytes, microglia, and blood vessels. Tumor cell interaction with these cells forms the tumor microenvironment (TME) which contributes to tumor growth, therapeutic resistance, and recurrence [1–7]. This complex TME needs to be considered in preclinical modeling of these tumors as the TME itself is relevant to not only tumor cell behavior and therapeutic response, but also the effect and/or toxicity of therapeutic drugs on the other healthy cells within and around the TME. Traditional in-vitro monoculture of tumor cells fails to faithfully recapitulate this complex TME and lacks the ability to accurately profile effect of potential therapeutic drugs on non-malignant TME cells. This leaves a large translational research gap in the in-vitro space for CNS tumors.

Traditionally, this gap has been filled by in-vivo animal models, however, this also comes with limitations [8–10]. Although the complex TME can be more faithfully represented using in-vivo modeling, there is an inherent species mismatch and complex brain cells such as astrocytes, microglia, and neurons, have all been shown to be significantly different across species especially between humans and mice [11–18]. Mouse models may also fail to model human-specific CNS toxicities [19], although current literature on this topic is sparse. Furthermore, reliable surgical implantation of tumors into the brains of animals is a complex surgical skill and is also heavily resource intensive. There has also been a lackluster track record of translation from promising therapy in animals to a viable therapy in human clinical trials [8–10]. These limitations and others have led the National Institute of Health (NIH) to release a new research directive in 2025 calling for a prioritization of novel scalable human-based modeling systems for preclinical testing. The idea behind these guidelines is not to entirely replace animal research but instead to augment preclinical testing, limiting the unnecessary use and suffering of animals and better model human biology.

To answer this call for novel human-based disease model systems we utilized a published and validated human planar neural organoid (PNO) which can be seeded with patient derived xenograft (PDX) cell lines, or fresh patient tissue, and maintained in culture [20–23]. We have previously shown that these 3-dimensional PNOs demonstrate self-assembly and can incorporate microglial progenitors [22]. The PNOs are developed from non-specified neural progenitor cells, which differentiate into radial glial cells, astrocytes, intermediate progenitors, and excitatory and inhibitory neurons [20]. Furthermore, PNOs can be consistently produced with minimal batch to batch variation making them a suitable modeling system that has already been utilized to study neuroinflammation and cellular toxicity [20, 22]. Human derived PNOs provide a direct species match and have critical components of the TME including astrocytes and microglia and can be used for reliable pre-clinical drug testing. In this study, we established, validated, and spatially profiled human PNOs seeded with pediatric diffuse midline glioma H3K27-altered (DMG) patient derived xenograft (PDX) cell lines and compared them with the matched method spatial transcriptomics of direct biopsies of patient tumor samples. We found that DMG cell lines spatially integrate into human neural PNOs. Tumor seeded PNOs recapitulate known genetic signatures from patient samples and have similar transcriptomic and proteomic landscapes. This complex neural PNO co-culture system is superior to tumor cell culture monoculture for normal cell off target toxicity screening and can be used for rapid drug screening and patient specific assays. Our results provide a crucial proof of concept for application of these PNO culture systems more broadly for preclinical investigation and drug screening.

## Methods

### Cell culture

iPSC-derived human Neural Precursor Cells (NPCs) from a male donor, (FCDI, Madison, WI) were maintained in complete STEMdiff Neural Progenitor Medium (NPM), (STEMCELL Technologies 05833). When thawing or passaging, 10 µM Y27632 (Rock inhibitor; Chemdea NC0407157) was included in the medium and removed day 1 post thawing/passaging. NPCs were cultured on Geltrex (Gibco A1413302) diluted 1:100 with DMEM/F12 (Gibco 11330032). Cells were cultured at 37 °C, 5% CO_2_ atmosphere and passaged at 95% confluent with StemPro Accutase (Gibco A1110501) diluted 1:1 with DPBS (Gibco 14190094). NPCs were assessed for expression of NPC markers SOX2, Ki67 and Nestin by immunofluorescence analysis and were used within 5 passages of the original cell banking.

iPSC-derived human iCell ECs (FCDI C1114) were cultured according to the supplier’s recommendations in VascuLife Basal Medium (LifeLine LL-0003) with VascuLife VEGF LifeFactors. Heat Inactivated FBS (HyClone SH30071.03HI) was added to the complete media at 10% in place of the supplied FBS. ECs were maintained on a coating of 3 µg/cm^2^ fibronectin (Sigma FC010). Cells were cultured at 37 °C, 5% CO_2_ atmosphere and passaged at 80% confluent with TrypLE Express (Gibco 12605028). Cells were used within 5 passages of receipt from the supplier.

iPSC-derived human iCell MSCs (FCDI R1098) were cultured in complete MSC medium, 0.25X Ham’s F-12 (Gibco 11765054), 0.75X IMDM (Gibco 12440046), 50 µg/mL Ascorbic Acid (Sigma A8960), 0.5X B-27 Supplement Minus Vitamin A (Gibco 12587010), 50 ng/mL bFGF (R&D Systems 233-FB), 0.05% BSA (Gibco 15260037), 1X GlutaMAX (Gibco 35050061), 450 µM MTG (Sigma M6145), 0.5X N-2 Supplement (Gibco 17502048), 50 ng/mL PDGF-BB (PeproTech 100-14B). MSCs were cultured on a coating of 5 µg/mL fibronectin (Sigma FC010) and 10 µg/mL collagen I, (Gibco A1048301). Cells were cultured at 37 °C, 5% CO_2_ atmosphere and passaged at 80% confluent with TrypLE Express (Gibco 12605028). Cells were used within 5 passages of receipt from the supplier.

DMG PDX lines (SF8628, SF7781, and SU-DIPG36) were graciously obtained from Dr. Rintaro Hasizhume and cultured according to published protocols [24, 25]. Briefly, lines were maintained in Neurobasal media (Gibco) supplemented with EGF (20ng/ml), FGF (20ng/ml) growth factors, N2 and B27 and 1% penicillin streptomycin (All from Thermo Fischer). Lines were grown as spheres for general cellular passaging and maintenance and plated onto attachment dishes or into PNOs for dosing experiments and PNO culture, respectively. All three lines carried the H3K27 mutation.

### PNO generation and culturing

Human neuroimmune PNOs were generated from NPCs, microglia (MG) (FCDI C1110), ECs, and MSCs as previously described [20]. Except for the microglia, cells were cultured per the cell supplier’s instructions prior to plating onto a PEG-based hydrogel substrate optimized for this application (proprietary formulation, Stem Pharm, similar to that described in [20–23]. iPSC-derived Microglia (FCDI R1131) were added directly to PNOs from cryopreservation. Generation of planar neural PNOs have previously been described [21–23]. The PEG-based hydrogel was polymerized in µ-Plate 96 Well 3D plates (Ibidi 89646) and equilibrated overnight in PBS and then in NMM prior to plating NPCs. NPCs, MSCs, ECs and MG were plated in serum free medium at the following time points; NPCs day 0 (25 K/well), MSCs and ECs day 3 (18 K and 1.8 K/well respectively), MG day 14 (12.5 K/well). PNOs were maintained in Neural Maintenance Media (NMM): DF3S (DMEM/F12 (Gibco 11330032) supplemented with 64 µg/mL L-ascorbic acid-2-phosphate magnesium (Sigma A8960), 14 ng/mL Sodium Selenium (Sigma S5261), 543 µg/mL Sodium Bicarbonate (Gibco 25080-094)) supplemented with 1X B-27 Supplement (Gibco 17504044), 1X N-2 Supplement (Gibco, 17502048), 1X GlutaMAX (Gibco 35050061), 1X MEM NEAA (Gibco 11140050), 1X Penicillin-Streptomycin (Gibco 15140122). NMM was supplemented with 5 ng/mL Heat Stable bFGF (Gibco PHG0367) day 0–5 and 100 ng/mL VEGF (R&D Systems 293-VE) day 3–13. Cultures were fed daily with 50% of medium removed and added back with fresh medium. The PNOs were cultured to day 21–28 post plating with NMM prior to the addition of DMG cells. 10,000 or 20,000 DMG cells (dependent upon experiment) were added per well and cultured for 7 days prior to supernatant collection, inhibitor dosing or immunofluorescence analysis.

### Supernatant analysis

Supernatant was analyzed from cultures with 10,000 DMG cells added, 7 days after DMG addition with the Human Cytokine/Chemokine Panel A 48-Plex Discovery Assay^®^ Array (HD48A) (Eve Technologies, Calgary, AB, Canada). Three biological replicates were assessed for each condition, supplements from two PNOs were pooled per biological replicate.

### Immunofluorescence analysis

PNOs were fixed with 4% paraformaldehyde in PBS for 1 h and stored in PBS at 4 °C. PNO samples were permeabilized and blocked with PBS containing 10% Donkey Serum (Sigma D9663), 0.2% TritonX-100 (Sigma T9284) and 0.02% Sodium Azide (Fisher S227I) (Antibody Incubation Solution, AIS) for 1 h. Primary and secondary antibodies were incubated in AIS. Anti-β3 tubulin (1:500 MAB1195, RnD Systems) and Anti-IBA1 (1:250 ab5076, Abcam) antibodies were incubated overnight at 4 °C and washed three times in PBS. Pre-conjugated Anti-H3K27M antibody (1:50, CST 85023 S), and AF-647 Donkey anti-Mouse (1:500, Thermo A32787) and AF-568 Donkey anti-Goat (1:500, Thermo A11057) were incubated overnight at 4 °C and washed three times in PBS. In separate wells, Anti-IBA1 (1:250, Wako 019-19741) was incubated overnight at 4 °C and washed three times in PBS. Donkey anti-rabbit AF555 antibody (1:500, Thermo A31572), and AF-647 pre-conjugated anti-GFAP antibody (1:250, Abcam AB302828-1001) and pre-conjugated AF-488 Anti-H3K27M antibody (1:50, CST 85023 S) were incubated overnight at 4 °C and washed three times in PBS. Images were captured on a Nikon AX R confocal microscope with a 20X objective. Image processing was performed in Nikon NIS-Elements. Maximum intensity projection images are presented.

### Sphericity quantification

Image processing was performed in Nikon NIS-Elements and Fiji. Maximum intensity projection images are presented. Microglia morphology analysis was performed on images taken on a Nikon AX R confocal microscope with a 10X objective. Analysis was performed using Fiji and 3D ImageJ Suite [26]. Microglia objects were filtered for object volumes of > 4,000 and < 60,000 in calibrated unit. Sphericity in calibrated unit was graphed and analyzed by one-way ANOVA with multiple comparisons.

### Spatial transcriptional analysis

PNOs were fixed with 4% paraformaldehyde in PBS for 1 h and stored in PBS at 4 °C and shipped to Acepix Biosciences (Union City, CA) for paraffin embedding and sectioning. 4 μm sections were prepared and arrayed on slides for spatial analysis. NanoString performed slide mounting and raw data processing and sequencing according to company protocols and standards. ROI selection was performed under the guidance of a neurosurgeon, neuropathologist, and neuro oncologist. ROIs were designed to encompass possibly useful possibly clinically relevant features and were distributed between regions of normal PNO and regions with significant tumor infiltration. A control set of ROIs from PNOs naive to tumor and cultured in separate wells to eliminate the possibility of cross contamination were also utilized. For downstream GeoMx sequencing and data processing analyses were purposeful mirrored closely to our previous work to make valid comparisons back to our patient samples [27]. Briefly, SCENIC regulon enrichment was conducted according to package standards and detailed elsewhere [27]. Differential expression was obtained using DESeq2 following all standard parameters [28]. RNA to protein correlation was conducted as previously described [27]. Spatial deconvolution utilized 3 different algorithms (CIBERSORTx, ReDeconv [29–31], and Spatial Decon) while signature comparison was conducted to previously published work [32] using methods detailed in our previous publication [27]. For PC clustering and UMAP visualization standard Seurat workflows (adapted for GeoMx data) were followed with cluster assignments being determined entirely within PC space (7 PC’s total) using SNN graphs and Leiden community detection.

### In-vitro dosing

For in-vitro dosing PNOs seeded with DMG cells were obtained from collaborators and dosed with p38 Map-Kinase inhibitor Adezmapimod (Selleck Chem), EGFR inhibitor Cyclopropanecarboxylic acid-(3-(6-(3-trifluoromethyl-phenylamino)-pyrimidin-4-ylamino)-phenyl)-amide (Sigma), Met-Kinase inhibitor SGX523 (Sigma), and HSP70 inhibitor (5.31067 sigma) at reported IC50 doses (given by manufacturer website). All drugs were solubilized in DMSO as recommended by manufacturer and equimolar DMSO was used for controls for each drug dosed. After 48 h of treatment PNOs were lifted from the hydrogel well plate using trypsin and light mechanical dissociation and filtered with 70 μm mesh to generate single cell suspension. Cells were then stained for flow cytometric analysis following lab protocols [4, 33] using antibodies CD45 (BioLegend), GFAP (BioLegend), H2K37m (Abcam) and Annexin V (BioLegend). After surface (CD45, annexin) and intra-cellular staining (GFAP, H3K27m) cells were resuspended in FACS buffer and analyzed on an Attune flow cytometer with analysis conducted in FlowJo.

### Statistics

Statistical analysis was conducted within bioinformatics analysis pipelines within packages were utilized if available. Statistics conducted on in-vitro dosing and for sphericity analysis were conducted in GraphPad Prism. For determination of statistical difference between patient and PNO deconvolution signature a Welch test for significance was conducted in RStudio with FDR corrected p-value of < 0.01 and a mean change of > +/- 0.1 being considered significant. PCA analysis and UMAP projection were conducted in Seurat following standard pipeline protocols [34]. For cytokine secretion comparison and dosing comparisons between drug conditions a 1-way ANOVA + Tukey’s multiple comparison test was conducted. All cartoon images were created using BioRender.

## Results

### DMG integrates into human neural PNOs, interacts with microglia and astrocytes, and drives cytokine secretion

**Fig. 1:**
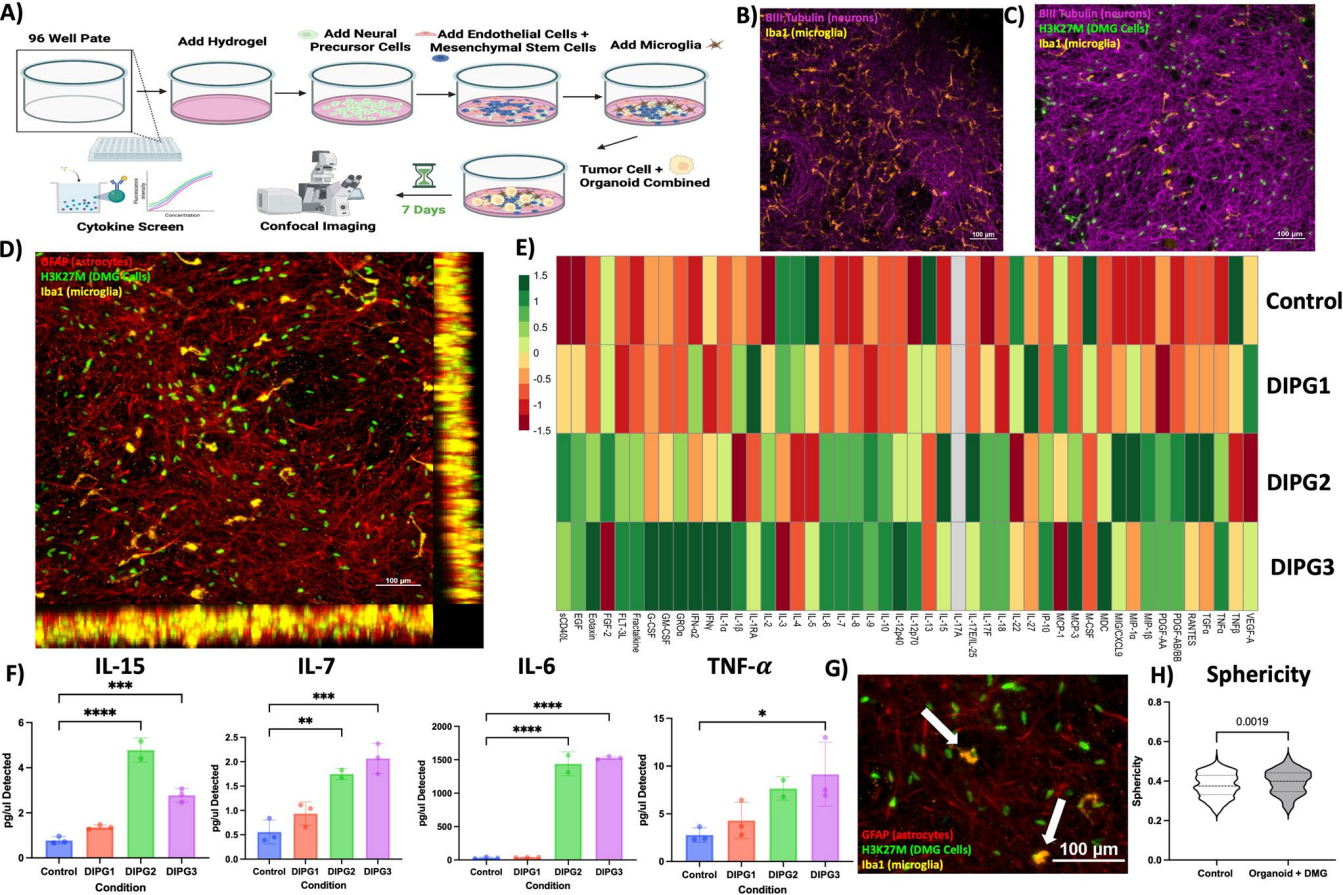
*DMG integrates into human neural PNOs*,* interacts with microglia and astrocytes*,* and drives cytokine secretion.* **(A)** Cartoon representing the experimental design of PNO construction and confocal imaging and supernatant based secreted cytokine screen. **(B)** 20X confocal image of PNO stained with β3 tubulin (purple) and Iba1 (yellow) without tumor cells present. **(C)** 20X confocal image of PNO stained with β3 tubulin (purple) and Iba1 (yellow) with H3K27m mutated (green) tumor cells present. **(D)** 20X confocal image with 3D reconstruction (side panel) of PNO + tumor cells stained with GFAP (red), H2K27m (green), and Iba1 (yellow). **(E)** Heatmap representing changes in cytokine secretion (normalized) across 48 cytokines and 3 PNOs integrated with separate DMG PDX lines versus a control non tumor seeded PNO. **(F)** Bar graphs representing pro-inflammatory cytokines across control and tumor seeded PNOs. **(G)** Zoom of 20X confocal image of tumor seeded PNO stained with GFAP (red), H3K27m (green) and Iba1 (yellow). **(H)** Violin plot of microglia sphericity measurement between control and tumor seeded PNO. * *p* < 0.05, ** *p* < 0.01, *** *p* < 0.001, **** *p* < 0.0001 `Grey bar indicates Non-Detected cytokine`

PNOs were generated on a PEG-based synthetic hydrogel as outlined in the methods section and previously described [21–23] (Fig. 1A). To evaluate if DMG tumor cells would integrate and disseminate into PNOs, as these cells do within the brains of patients, we first attempted a co-culture with 3 different DMG PDX cell lines added to the PNOs on day 21 of PNO culture (Fig. 1A). To follow the cellular interactions between tumor cells and PNO cells we employed confocal microscopy and immunofluorescence to visualize PNOs without (Fig. 1B) and with (Fig. 1C) the addition of tumor cells (H3K27m, green), microglia (IBA1, orange), or neurons (β3 tubulin, magenta), or astrocytes (GFAP, red) (Fig. 1B & C). Results demonstrated that DMG cells integrate and invade through the layers of the PNO much like is seen in histological analysis of these tumors, something that is impossible in a tumor cell monolayer culture system (Fig. 1D). To probe the functional consequences of introducing tumors into the PNOs we conducted a multiplexed cytokine screen from the secreted media in standard PNO cultures or PNOs that had been integrated with tumor cells for 1 week. Across the 48 analyzed cytokines we saw both broad and cell line specific increases and decreases of cytokines released in control PNOs vs. tumor seeded PNOs (Fig. 1E). DMGs are known for significant interpatient tumor heterogeneity, this heterogeneity was also captured in the PNO model where the three different cells lines had different cytokine signatures (Fig. 1E). Overall, the PNOs had a strong pro-inflammatory reaction to the addition of the tumor cells with significant increases in secretion of *IL-15*, *IL-7*,* IL-6*, and *TNF-α* (Fig. 1F, *n* = 3 technical replicates). Although we did see a significant increase in pro-inflammatory cytokine secretion the anti-inflammatory cytokine *IL-10* was also increased mimicking the complex TME present in patient tumors which can at times be both anti and pro- inflammatory [35–40] (Sup Fig. 1A). At the cellular level we observed numerous instances of activated and more sphere-like microglia either directly contacting or even engulfing tumor cells as demonstrated by H3K27M antibody labeling within microglia (Fig. 1G & H, Sup Fig. 1B & C).

We believe that these cytokine changes are likely a combinatory result from secretions of the tumor cells as well as a pro-inflammatory response from the microglia as evidenced by increase *IBA1* staining and sphericity (Sup Fig. 1B & C). In previous work with PNOs in response to neurotoxic chemicals we noticed a similar *IBA1* and pro-inflammatory cytokine spike [20]. These data demonstrate that DMG tumor cells can integrate into, be maintained within, and potentially impact, human PNOs.

### GeoMx spatial sequencing demonstrates different regional differential expression and regulon enrichment between control and tumor PNOs

**Fig. 2:**
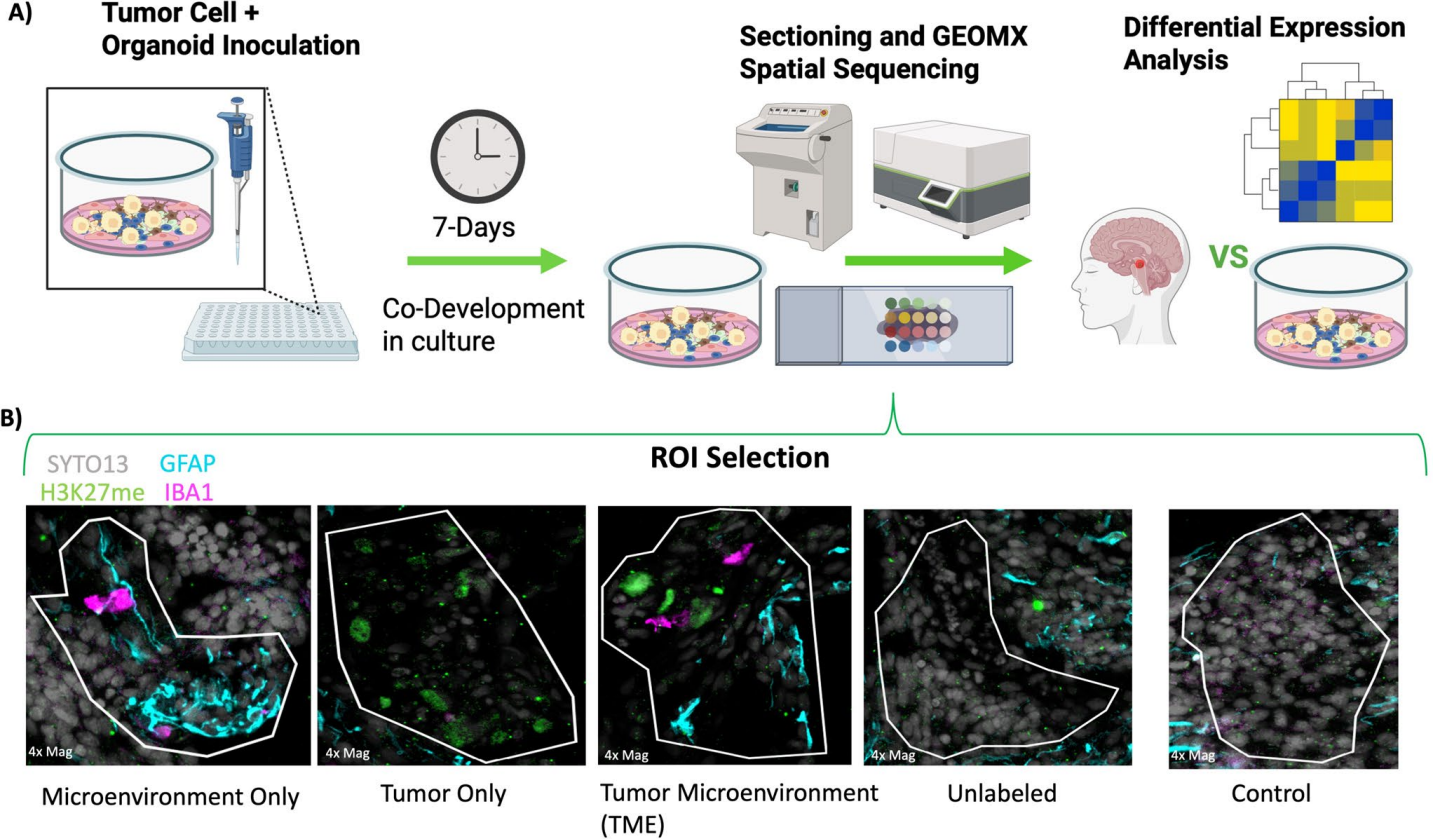
*GeoMx processing and ROI selection for control and tumor seeded PNOs.* **(A)** Cartoon depicting the experimental workflow of processing PNOs for GeoMx sequencing. **(B)** Representative ROI images which were selected for the GeoMx experiment

To process control and tumor PNOs for GeoMx, 3 separate DMG lines (SF8628, SF7781, SU-DIPG36) were integrated into the PNOs by dissociating the tumor cells to a single cell suspension and plating 10,000 tumor cells onto the PNO. Once adhered the PNOs were stained with a nuclear stain (Syto 13-grey), a microglial marker (IBA1-pink). an astrocyte marker (GFAP-blue) and a tumor cell marker (H3K27m-green) and imaged for region of interest (ROI) selection (Fig. 2A). A control ROI of unlabeled cells was selected only from a subset of standard PNOs never exposed to tumor cells (entirely separate wells preventing cross contamination) and sequenced identically to the tumor seeded PNOs (Fig. 2B). From PNOs with tumor cells integrated, ROI’s representing the PNO microenvironment consisting of microglia and astrocytes without tumor cells present (Microenvironment Only), tumor cells within the PNO (Tumor Only), the PNO microenvironment with tumor cells present (TME) and remaining PNO cells that were unstained (Unlabeled), were selected. These ROIs were used for region marking through the analysis.

**Fig. 3:**
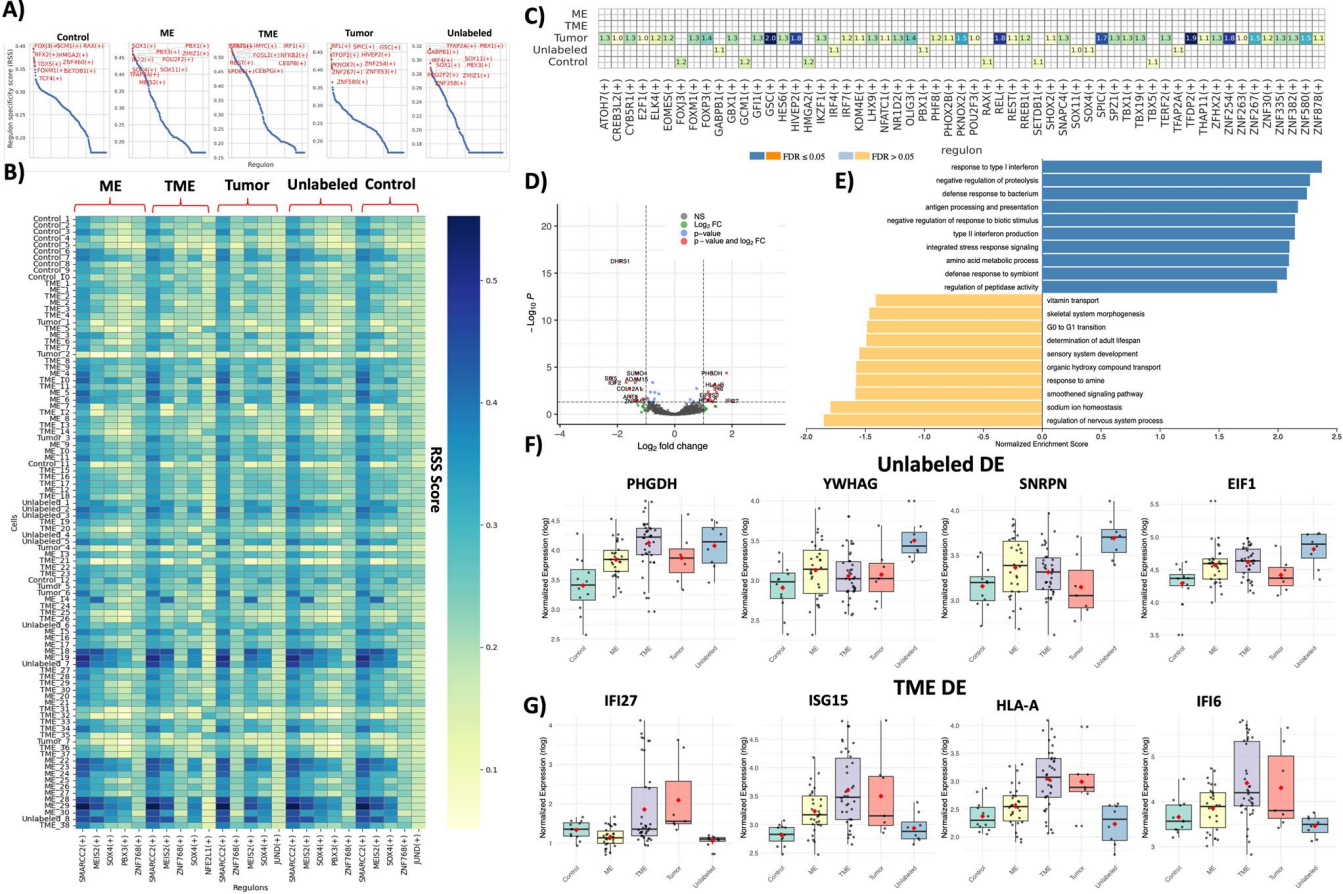
*GeoMx spatial sequencing demonstrates significantly different regional differential expression and regulon enrichment between control and tumor PNOs*. **(A)** SCENIC RSS plot for all selected ROIs in the GeoMx analysis. **(B)** Heatmap displaying the RSS value of the 25 top scoring regulons across all analyzed ROIs. **(C)** Heatmap displaying the Z score of the specific regulon and which region the significant enrichment belongs to. **(D)** Volcano plot displaying DE gene expression at the RNA level of TME ROIs compared to control ROIs. **(E)** GSEA results displayed for DE genes between TME ROIs and Control ROIs. **(F)** Box plot displaying the top 5 DE genes in unlabeled ROIs. **(G)** Box plot displaying the top 5 DE genes in TME ROIs

Spatial transcriptomics was used to understand the effect of cellular interactions of the tumor cells with the PNO cells. Using SCENIC regulon enrichment [41], we uncovered regional variation within the RSS (regulon specificity score) of the PNOs across all defined regions with some regions sharing regulons as well as some having unique regulon enrichment (95 ROI’s total across 5 defined regions) (Fig. 3A & B, Sup Fig. 2). A large majority of the variation was found in the tumor regions of the PNOs indicating that tumor cells had a highly significant impact on the regulon expression variation within the PNO, even though they made up roughly 5% of the total cells within the PNO itself (Fig. 3C). To visualize this change we compared differential expression at the RNA level using DESeq2 [28] from the control PNOs to TME regions in tumor PNOs and found 4370 genes total which were subdivided into highly significant (> 2 log2fold change & p-value < 0.05) sets for up and down regulation of gene expression (Fig. 3D). GSEA analysis across the significant DE genes signaled a major upregulation and shift in the inflammatory response of the PNO with enriched terms such as type 1 and 2 interferon response, antigen processing and presentation, integrated stress response signaling. (Fig. 3E). There was also downregulation of terms hinting at a slowing of the normal developmental process such as regulation of nervous system process, and sensory system development indicating that the tumor cells may be impacting the PNO normal growth patterns, however, these comparisons did not survive FDR correction at the 0.05 level (Fig. 3E). To further visualize the differential expression at the regional level, the top 4 DE genes from the unlabeled regions, which come from PNOs that have tumor cells in them but contain no tumor cells within the region isolated for GeoMx, and the TME regions were examined (Fig. 3F & G). These expression patterns demonstrate the enrichment of immune related genes in the TME (*HLA-A*,* IFI27*,* IFI6*) versus standard growth regulatory genes in the PNO itself (*EIF1*,* SNRPN*,* PHGDH*). These results highlight the impacts of tumor cell seeding within human neural PNOs and demonstrate that regional differences in expression are present.

### Human neural PNOs mirror transcriptomic signatures from patient tumor samples

**Fig. 4:**
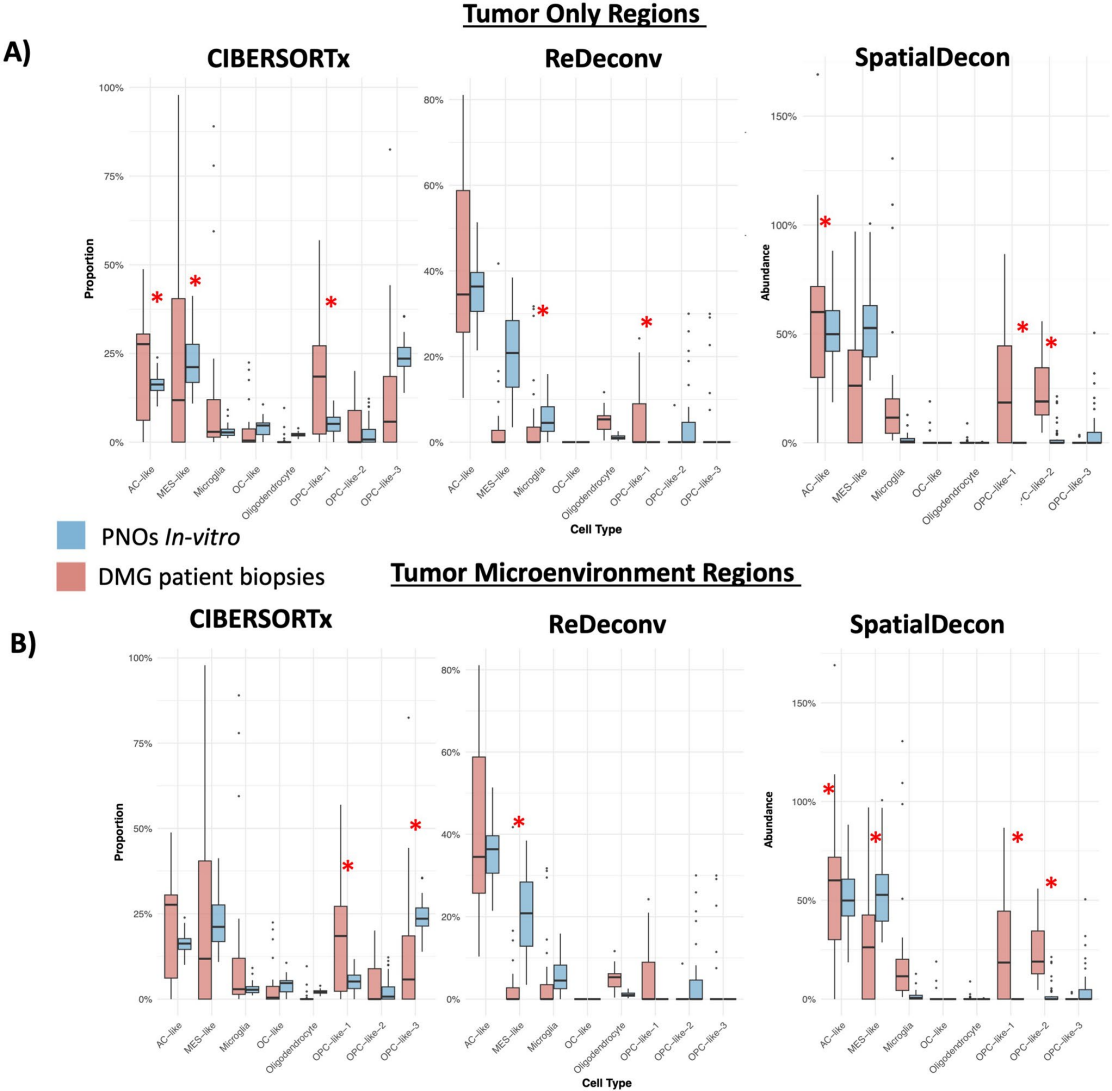
*Human PNOs mirror transcriptomic signatures from patient tumor samples.* **(A)** Box plots displaying cell signature comparisons across 3 deconvolution algorithms with PNO data in blue and patient biopsy data in red. **(B)** Box plots displaying cell signature comparisons across 3 deconvolution algorithms with PNO data in blue and patient biopsy data in red (right). Single red star indicates significance of the comparison detailed in results

To understand how closely the human neural PNOs seeded with tumor cells model human DMG, we conducted a comparison between identical GeoMx sequencing of DMG patient’s tumor form our previously published work [27]. Using established transcriptomic signatures/metaprograms from [32] we conducted deconvolution and comparison across matched GeoMx regions (i.e. regions containing only tumor cell from patients’ biopsies and regions containing only tumor cells from PNOs). Comparison of tumor cell ROIs from patient samples and tumor PNOs identified significant signature differences (welch test FDR corrected p-value of < 0.01 and a mean change of > (+/-) 0.10, calculated over sum of 95 total ROI’s in PNOs and 85 in patient biopsies) in 3/8 total signatures tested using CIBERSORTx [29] and 2/8 total signatures tested using ReDeconv [30] and 3/8 total signatures tested using SpatialDecon [31] (Fig. 4A). To test the impact of other cell types on the signatures we included TME regions and repeated the above comparison. When comparing TME ROIs from patients and TME regions from PNOs results were similar with 2/8 significantly different comparisons using CIBERSORT and 1/8 significantly different comparisons using ReDeconv and 4/8 significantly different comparisons using SpatialDecon (Fig. 4B). Across both ROIs the PNOs faithfully recapitulated between 50% and 87.5% of the transcriptomic signatures found in human samples validating their use as a reliable model system. Although there was much agreement there were also differences, likely due to the nature of the surrounding cells being a stem cell-based PNO and not a developed brain. For example, the transcriptomic signatures such as the S and G2M cell cycle signatures which were consistently enriched in PNOs relative to patient samples (Sup Fig. 3A) suggesting that PNOs are still quite proliferative in general when compared to developed brains even among pediatric samples. Deconvolution signatures were also compared solely across PNO ROIs which demonstrated mostly stable region-specific expression (Sup Fig. 3B).

### Human neural PNOs demonstrate similarities at both the RNA and proteomic levels compared to patient biopsy samples:

**Fig. 5:**
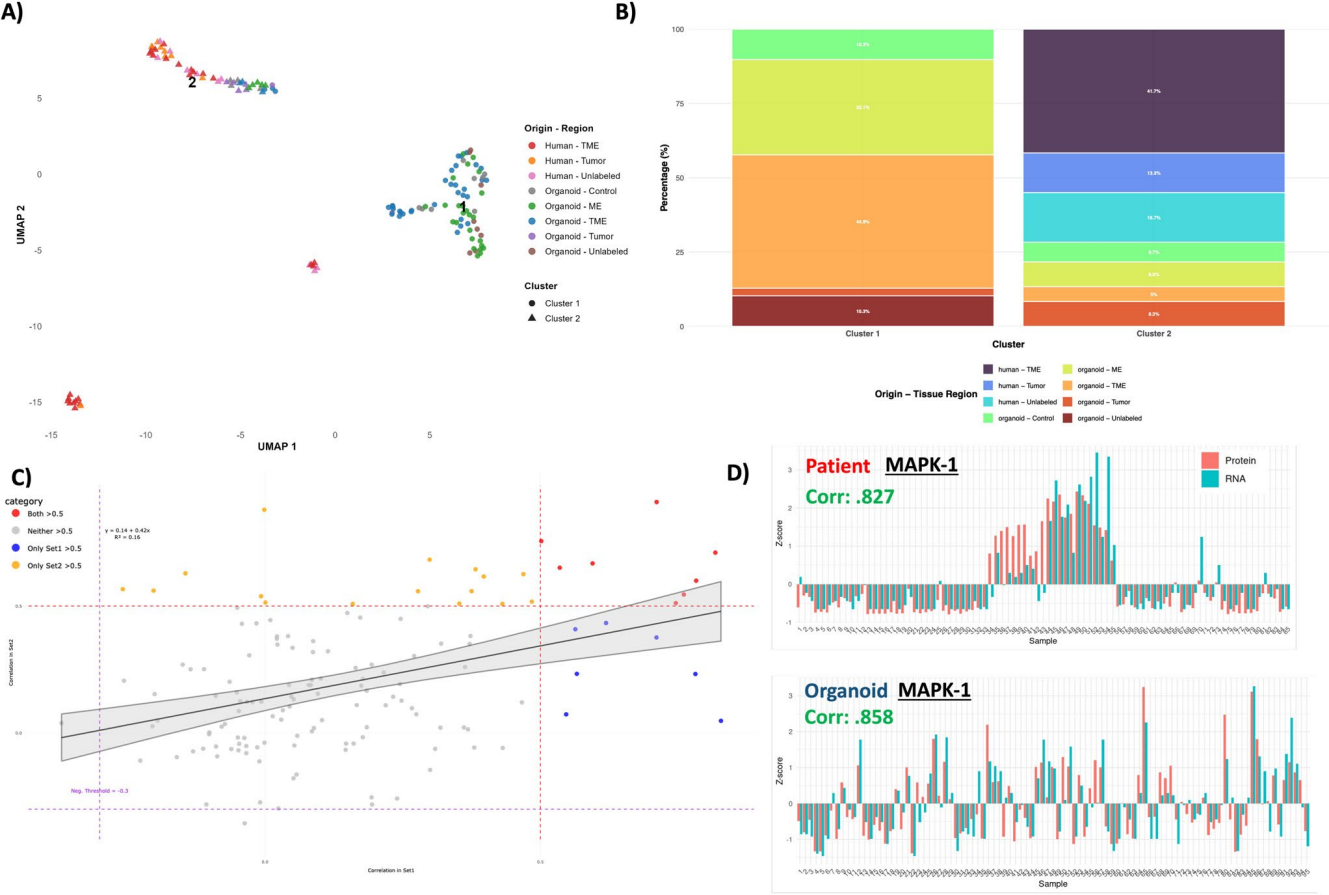
*Human PNOs demonstrate similarities at both the RNA and proteomic levels compared to patient biopsy samples.* **(A)** UMAP plot visualizing annotated clusters across the patient biopsy data and the tumor seeded PNO data. **(B)** Stacked bar plot representing the contributing regions detected in each UMAP cluster from either patient biopsy data or the tumor seeded PNOs. **(C)** RNA to protein correlation plot displaying the calculated correlation value across the patient biopsy dataset (set 1) and the tumor seeded PNO dataset (set 2). Legend indicated correlation level between RNA and protein while each dot represents an RNA and protein pair. **(D)** RNA to protein correlation plot produced for MAPK with correlation values across both patient biopsy and PNO datasets. (In Fig. 5A/B Organoid = PNO, Human = Patient Biopsy)

To further understand the similarities and differences between tumor PNOs and patient samples we performed principle component analysis (PCA) using an SNN graph and Leiden community detection on GeoMx RNA data from our tumor PNOs and patient samples. For visualization we employed UMAP visualization from the standard Seurat workflow which is commonly adapted for handling of GeoMx data (Fig. 5A). UMAP demonstrated a PNO only cluster and combined patient and PNO cluster along with 2 small patient-only clusters not reaching the level of significant difference (tested from resolution 0.3–0.6) (Fig. 5A). Cluster 1 (78 ROIs) is entirely organoid, while Cluster 2 (60 ROIs) contains all 43 human ROIs plus 17 organoid ROIs. Results demonstrate that the majority of PNO samples form their own cluster, but a subset co-cluster with the patient biopsy samples, indicating partial transcriptomic similarity between the two (Fig. 5A). To further visualize the transcriptomic makeup of the clusters we analyzed the proportion of cells coming from each ROI across our human and PNO samples and projected their location within either cluster 1 or 2 (Fig. 5B). All PNO ROIs except the Unlabeled and Control ROIs were present in the mixed human and PNO cluster (Fig. 5B). The breakdown of the general cell type (patient vs. PNO) as well as patient vs. PNO enrichment terms were also examined (Sup Fig. 4A & B). As another measure of similarity, we also visualized correlation between RNA and protein levels derived from GeoMx proteomics data (see methods and our previous work [27]). We found no significant difference in the overall correlation between RNA and protein in tumor PNOs when compared to patient biopsy samples (Sup Fig. 4C). On a gene-by-gene basis when comparing PNO’s to patient samples, we saw a large agreement in RNA to protein correlation with most genes showing no strong correlation in both the PNO and patient samples (Fig. 5C). We also examined a set of genes which shows very strong correlation between RNA and protein in both the PNO and the patient samples. One of these genes that is highly clinically relevant as a target in many brain cancers is the MAP-Kinase family [42]. We visualized the protein probe for MAPK1 across our patient biopsy and PNO dataset demonstrating a strong correlation in both patient samples and tumor seeded PNOs (calculated over sum of 95 total ROI’s in PNOs and 85 in patient biopsies) (Fig. 5D). For further confirmation, clustering using only 2 of the 7 detected PC’s also demonstrated 2 unique clusters both containing regions from PNO and patient biopsy samples (Sup Fig. 4D). These concurrent correlations likely indicate useful drug targets that are seen in patient samples and can be faithfully modeled within tumor PNOs establishing a valid system for preclinical off target toxicity drug screening. This data also demonstrates that although there are unique transcriptomic signatures related to the PNOs themselves, there is broad transcriptomic correlation between patient samples and PNOs at RNA and protein levels.

### Toxicity testing in human neural PNOs reveal cell type specific toxicity not captured in pure tumor cell monolayer culture

**Fig. 6:**
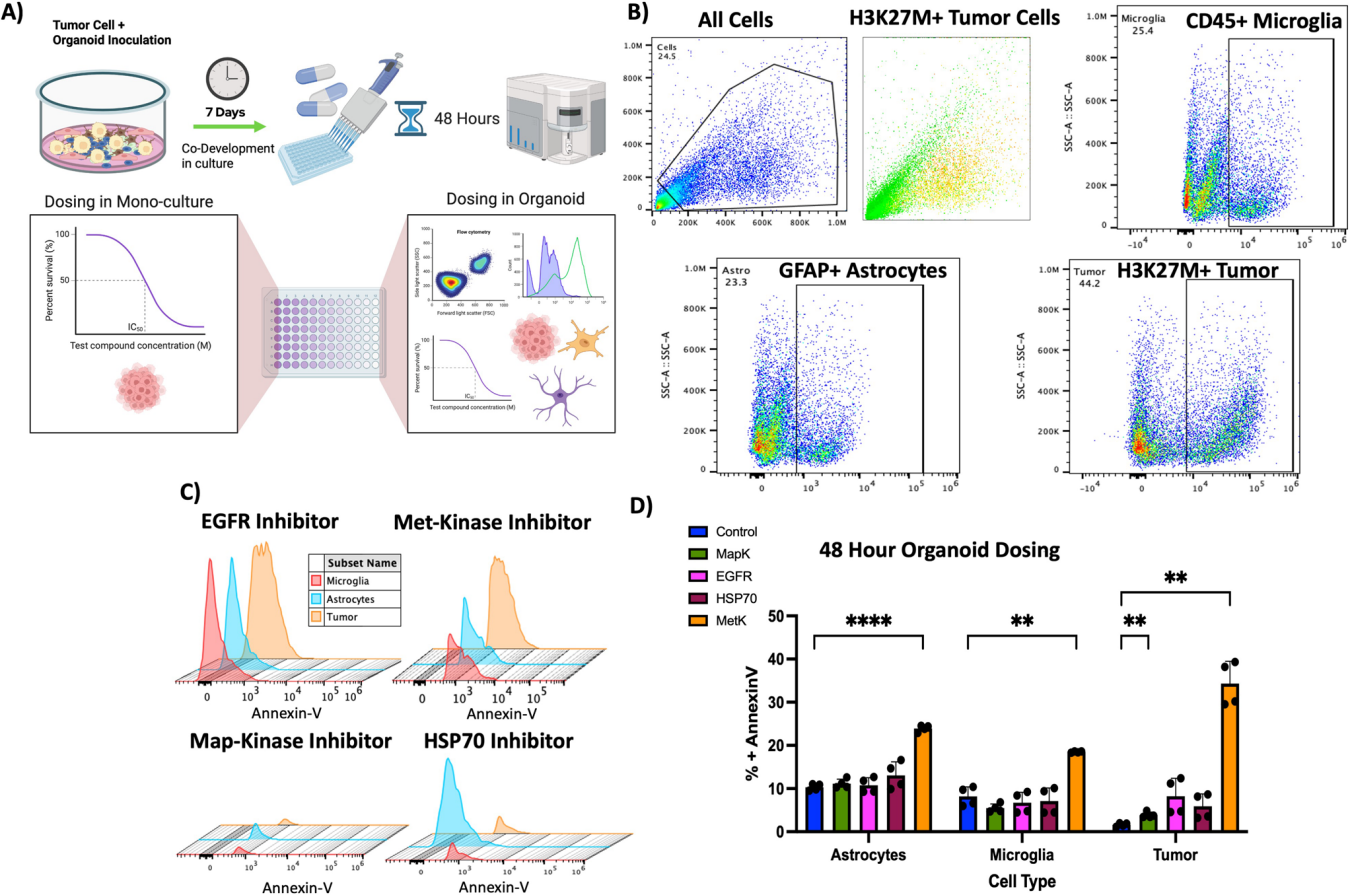
*Toxicity testing in human PNOs reveal cell type specific toxicity not captured in pure tumor cell monolayer culture.* **(A)** Cartoon depicting the experiment workflow of PNO drug dosing. **(B)** Representative FACS gating plots for the drug dosing experiment. **(C)** Annexin-V staining plots for each cell population across all drugs tested. **(D)** Bar chart displaying the % Annexin-V staining for all cell populations and control PNOs across experimental replicates (2) and technical replicates (2). * *p* < 0.05, ** *p* < 0.01, *** *p* < 0.001, **** *p* < 0.0001

A critical step in cancer targeting drug discovery is understanding the toxicity of the therapeutic compound to normal cells. Thousands of compounds have been found to kill cancer cells in a monolayer in-vitro which go on to cause unacceptable levels of normal cell toxicity when tested on in-vivo animal models. Nowhere is this toxicity more important than in the brain where off-target therapeutic effects can quickly become lethal complications. Traditional monoculture methods require multiple assays and repeated experiments to test toxicity across different cell types, and rely on the premise that these cell types act the same in isolation as they do in a surrounding microenvironment structure. This leads to unnecessary researcher time wasted and a large reliance on animals as a first pass of toxicity testing which many would consider to be often costly and inhumane [43–45]. To illustrate the application of tumor PNOs in this context we performed a toxicity study on our PNOs highlighting their advantages in speed and profiling of species matched off target toxicity (Fig. 6A). For toxicity testing, we selected 4 compounds which have already been pharmacologically profiled in traditional monoculture settings in brain cancer [46–49]. We applied these compounds at the characterized IC50 dose to profile tumor cell and surrounding microenvironmental cell toxicity within our PNOs. Flow cytometry was conducted to fluorescently label tumor cells, microglia, and astrocytes using H3K27m, CD45, and GFAP as cell specific markers (Fig. 6B). Using Annexin-V staining we quantified the level of toxicity on each cell population during drug treatment (detailed in methods) and found the MAP-Kinase inhibitor compound (dosed at 5 μm) to exhibit little toxicity to any cell type. Two compounds the EGFR inhibitor (dosed at 21nM), and the Met-Kinase inhibitor (dosed at 40nM) had tumor cell toxicity as well some as toxicity to normal cells. One compound, the HSP70 inhibitor (dosed at 5 μm), was selectively toxic to the astrocyte population (Fig. 6C). Comparing these compounds to vehicle control the Met-Kinase inhibitor showed the most significant toxicity to tumor cells (*p* < 0.01, 1-way ANOVA + Tukey’s multiple comparison test, *n* = 4 technical replicates) however it also exhibited significant toxicity to both astrocytes and microglia (*p* < 0.001 and *p* < 0.01, 1-way ANOVA + Tukey’s multiple comparison test, *n* = 4 technical replicates) although to a lesser extent than to tumor cells (Fig. 6D & E). Interestingly, we also found a much higher level of Annexin-V staining in vehicle control conditions for astrocytes and microglia when compared to the same level of vehicle control dosing in tumor cells. With all compounds being solubilized in DMSO (recommended by manufacturer) it is possible that normal cells have less tolerance for DMSO than tumor cells do. These results establish that tumor PNOs are feasible and valid preclinical tool in testing compound toxicity as well as establishing tumor cell lethality for experimental drugs. The information gleaned from this model system is superior to monoculture testing and is experimentally feasible on a large scale and within a short time window.

## Discussion

Even with extensive research in the field of DMG and other CNS tumors, a true therapeutic breakthrough has been elusive. Current pre-clinical research efforts rely heavily on initial in-vitro cell culture and subsequent animal models for cancer cell target testing and off target toxicity screening. While these models will continue to be one of the foundational steps for medical research there is little debate that their translatability needs significant improvement [8–10]. Our study provides a proof of concept for a novel human-based resource that can bridge this translational gap and relieve the pre-clinical safety testing burden currently placed solely on animal models. Using scalable high-throughput drug screening methods, innovative new compounds can be tested for both anti-cancer efficacy and off-target toxicity first in a species matched system and then advanced to in-vivo pre-clinical models only if they show promise. Our results indicate this experimental strategy is feasible and that tumor PNOs faithfully represent patient samples at both transcriptomic and proteomic levels. Furthermore, tumor PNOs display region specific complexity something also seen in patient biopsies that is impossible to obtain in standard monoculture [50, 51]. This research lays the groundwork for more complex and mechanistic interrogation of DMG and its interaction with the tumor microenvironment. Indeed, our imaging results visualize microglia in close proximity and possibly interacting with DMG cells (Fig. 1G). Furthermore, resulting cytokine and morphological changes of the microglia indicate strong activation in response to tumor cells both locally and regionally (Fig. 1F & H). Further studies possibly using Xenium high resolution spatial architecture or single cell genomics could more accurately profile single cell traits than our GeoMx data. This model system also allows for controlled in-vitro perturbations, can more thoroughly interrogate these microenvironment interactions.

A growing body of literature has identified PNOs as useful in many disease types from cancers to diseases of the retina and numerous other groups have provided validation of PNOs in other brain tumors such as glioblastoma (GBM) [52–55]. We chose to investigate DMG in our PNOs due in part to its difficulty to modeling in-vivo and its dismal overall survival rate and need for therapeutic innovation. In addition, we recently published the spatial transcriptomics and proteomics of DMG patient’s tumor samples that laid the foundation for proper comparison of the tumor PNOs with the human disease [27]. This work builds on a recent study demonstrating that that similar neuroimmune-competent brain PNOs interact with tumor cells and specifically cause an observable microglial response [56]. We validate these conclusions and provide critical context as we can, for the first time, tie direct transcriptomic signatures within the PNO to actual tumor biopsies obtained from patients. Together these studies indicate that PNOs are robust and dependable models for DMG and fill a critical need in the preclinical research space which cannot be filled solely by in-vivo mouse modeling.

Although some organoids are much larger (fully spherical) and kept in culture for months to study neurodevelopmental dynamics, our PNOs are grown in 96 well plates and take weeks not months to generate (see methods). These advantages allow experiments to be scaled up and optimized for high-throughput drug screening. Indeed, one of the promising aspects of PNO use in general for disease modeling is their customizability and the ability for them to be modified to suit experimental or model system needs.

Although research on these organoid model systems is robust and encouraging, they are not without their own limitations that must be considered, as is the case with any model system. The culture and construction of PNOs is considerably more complex and specialized than cancer cell monoculture and requires specific training, resources, medias, growth factors, and incubator setups. Because of these culture conditions, and specifically the heavy use of growth factors and media supplements, we risk altering the transcriptomic and functional landscape of the tumors by removing them from a developed brain (adult or child) and putting them into somewhat primitive stem cell-based environment. In tumors such as GBM, which have been shown to have a large stem cell-like compartment, it’s possible that we will unequally bias towards growth of these types of cells which may not be found in that concentration in actual tumors.

Future development of this model system will rely on PNOs that are able to be produced consistently. Our human neural PNOs have been demonstrated to have high uniformity between individual PNOs transcriptionally and have already been used in applications of neurotoxicity testing [20, 21]. Data from this study demonstrate that their spatial signatures are stable with most variation being caused by tumor regions (Fig. 3C) not changes across individual PNOs themselves. Furthermore, as the use of PNOs becomes more common in pre-clinical and translational settings, it is of utmost importance to define context of use for each system as different methodologies will serve different purposes. To successfully launch any organoid model as a bridge for pre-clinical testing, the various groups working in this space need to coalesce towards common terminology on what constitutes an “organoid”. For now, we defer to a consensus nomenclature published in Nature in 2022 [57] by leaders in the field which would classify this PNO as an organoid because it is not simply an assembly of fully differentiated cells. The astrocytes, radial glia, intermediate progenitors, and neurons present in the system are derived from neural precursor cells and differentiate and interact with each other during the growth/assembly process. These PNOs are 3-dimensional, grow on a complex hydrogel substrate and have been shown to exhibit self-assembly, something not seen in traditional co-culture techniques [22].

As spatial genomics technology has progressed pass GeoMx, which is a targeted regionally specified bulk analysis, new offerings from 10x and Nanostring alike have demonstrated the ability to profile down to single cell resolution while maintaining a spatial grid for location-based context. This technology has already proven to be incredibly useful in the context of gliomas and other tumors (reviewed here by our group [58]) and would be a logical follow up step to more directly test many of the claims made with this analysis. Indeed, direct testing of the transcriptomic profile and longitudinal development of astrocytes that are within a certain distance from tumor cells or are more separated from tumor cells is a provocative question PNO’s would be well equipped to solve.

Modeling a human disease which is often multifactorial in nature is a demonstrably difficult task and will most likely require the integration of several different model systems. The challenge is to accomplish this task in the most cost and time effective manner to advance science for patient benefit. No model system by itself will ever be perfect and PNOs at this stage are not ready to fully replace in-vivo animal work. We provide validation and functional data that our tumor PNO system is superior to the in-vitro monoculture and is a useful supplement to reduce the burden on animals and enhance the testing and eventual translatability of novel compounds. In an era where considerable progress has been made in many cancers, CNS cancers lag behind, certainly due in part to the difficulty in modeling them in the lab. Tumor PNOs represent a new tool in the belt of researchers for a set of diseases in which patients are desperate for innovation and bystander local toxicity is of very high consequence.

## Supplementary Information

Below is the link to the electronic supplementary material.

Supplementary Material 1: Fig. 1: A) Bar chart showing anti-inflammatory cytokine IL10 cytokine secretion across experimental conditions. B & C) Confocal images used to calculate microglia sphericity 20X (Iba1 stained in orange).

Supplementary Material 2: Fig. 2: Heatmaps of regulons broken out by ROI showing top 10 enriched terms.

Supplementary Material 3: Fig. 3: (A) Deconvolution results across 3 different algorithms displaying significance for cell cycling signatures across both patient biopsy (human) and tumor seeded PNO (PNO) groups. (B) Box plot displaying the same signature comparison conducted above but across PNO ROIs only.

Supplementary Material 4: Fig. 4: (A) bar chart showing percentage of ROIs within each cluster from either patient biopsies (human) or tumor seeded PNOs (PNO). (B) Enriched GSEA terms across databases (left) and by each experimental group (color). (C) Overall comparison between total correlation value of RNA to protein when all pairs are combined together between patient biopsy samples and tumor seeded PNOs. (D) PCA only based clustering of the patient biopsy sample vs. PNO using PC 1 and 2.

## Data Availability

Raw counts data from Novogene has been uploaded to Zenodo and will be released upon publication of the article DOI: [10.5281/zenodo.16915253](10.5281/zenodo.16915253) . Further information about the datasets used and/or analyzed during the current study are available from the corresponding author on reasonable request.
