# Supplementary figures and images for: Human neural organoid modeling of diffuse midline glioma captures the complexity of patient tumors
Source: J Neurooncol. 2026 May 7;177(3):136. doi: 10.1007/s11060-026-05515-5 (PMC13152894; doi:10.1007/s11060-026-05515-5)

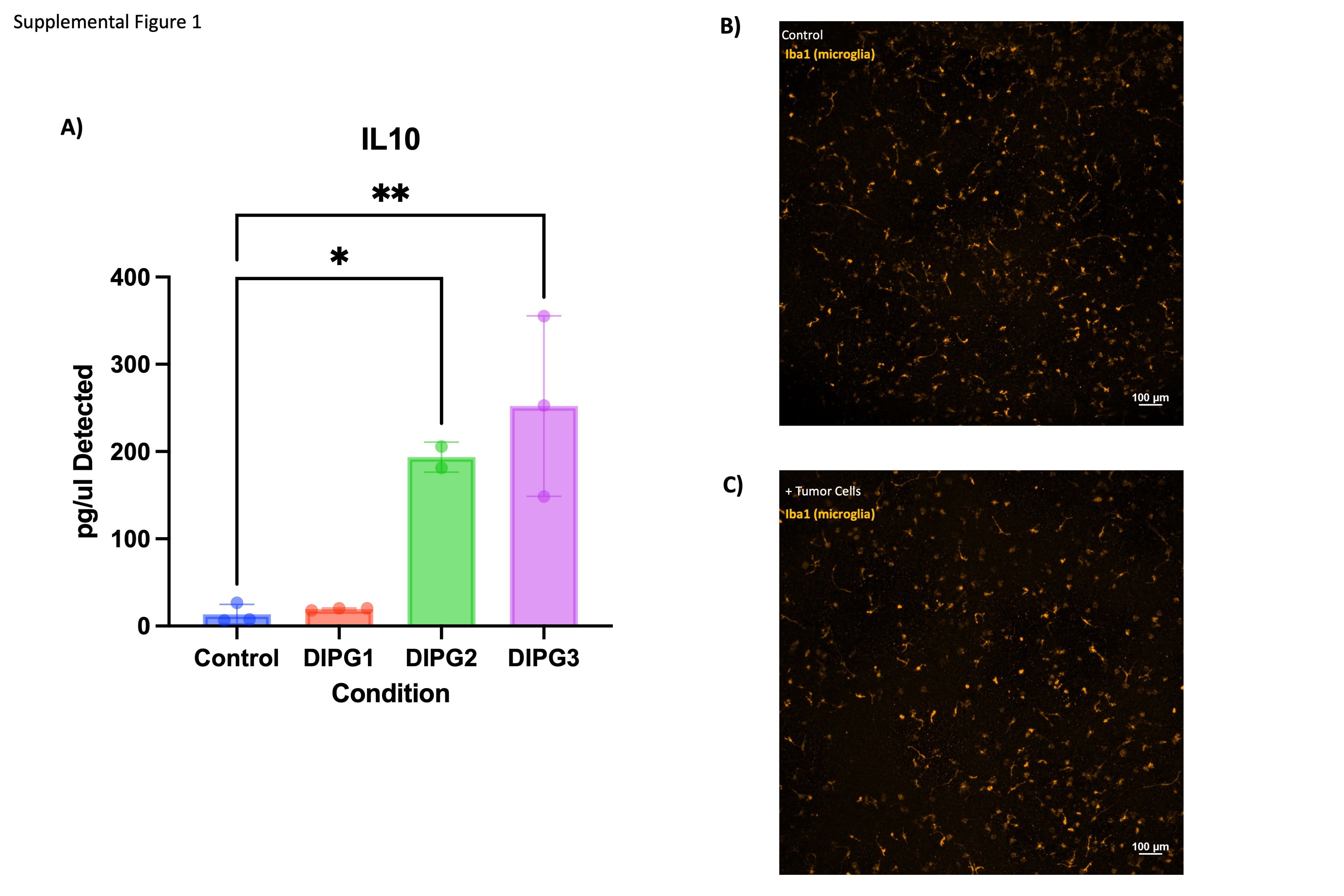

Supplement: Supplementary file 1 — Supplementary Material 1: Fig. 1: A) Bar chart showing anti-inflammatory cytokine IL10 cytokine secretion across experimental conditions. B & C) Confocal images used to calculate microglia sphericity 20X (Iba1 stained in orange). [file 11060_2026_5515_MOESM1_ESM.jpeg]

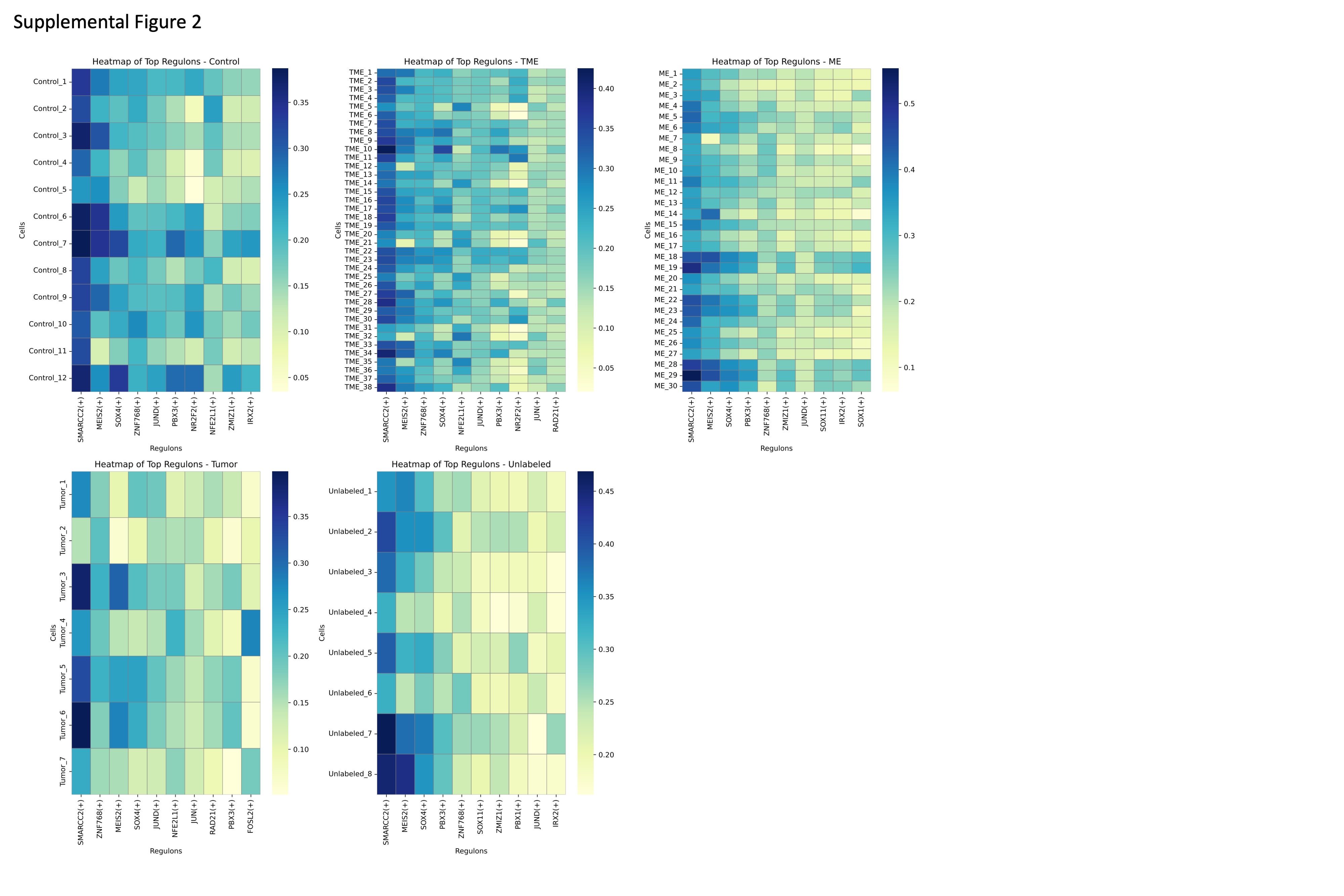

Supplement: Supplementary file 2 — Supplementary Material 2: Fig. 2: Heatmaps of regulons broken out by ROI showing top 10 enriched terms. [file 11060_2026_5515_MOESM2_ESM.jpeg]

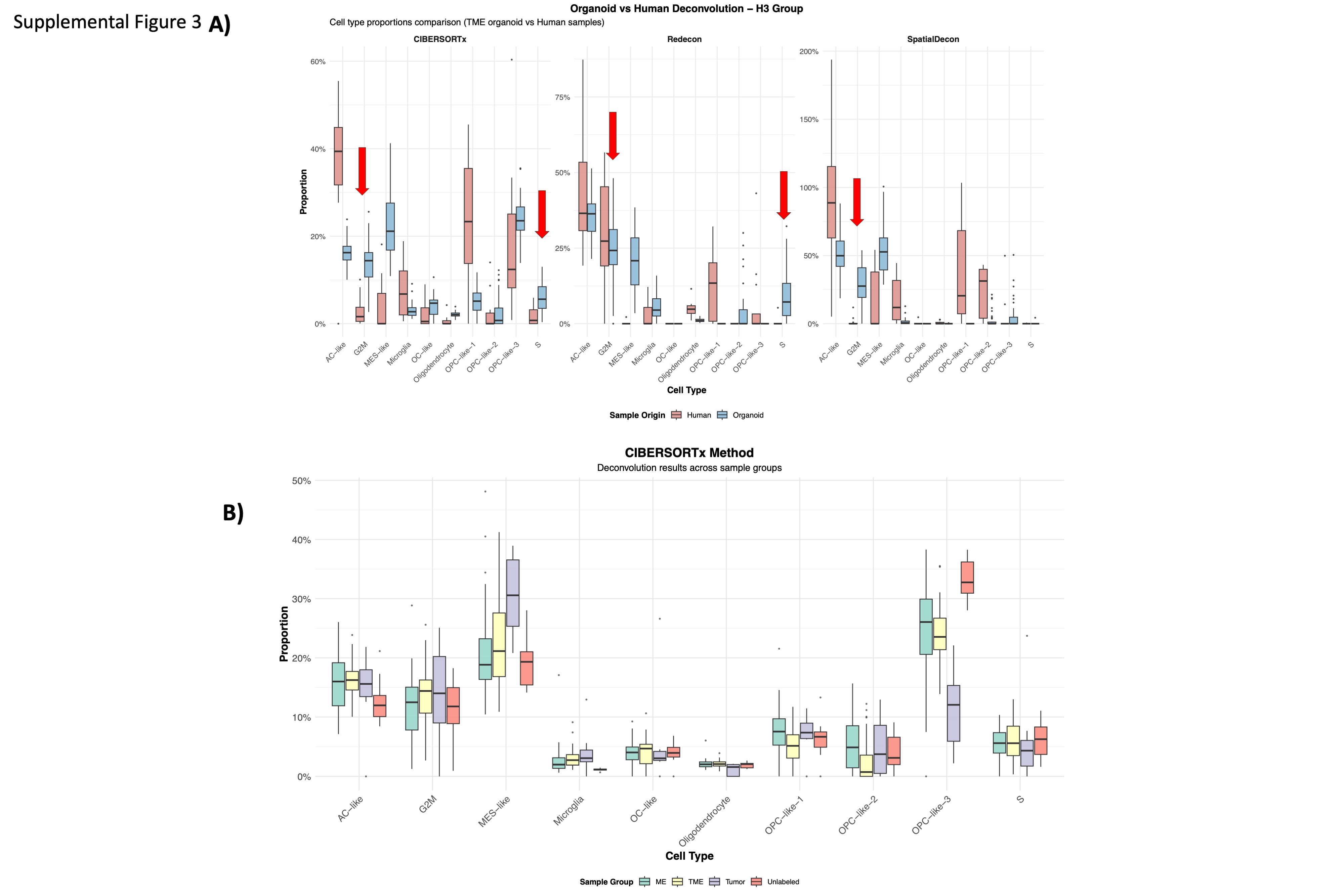

Supplement: Supplementary file 3 — Supplementary Material 3: Fig. 3: (A) Deconvolution results across 3 different algorithms displaying significance for cell cycling signatures across both patient biopsy (human) and tumor seeded PNO (PNO) groups. (B) Box plot displaying the same signature comparison conducted above but across PNO ROIs only. [file 11060_2026_5515_MOESM3_ESM.jpeg]

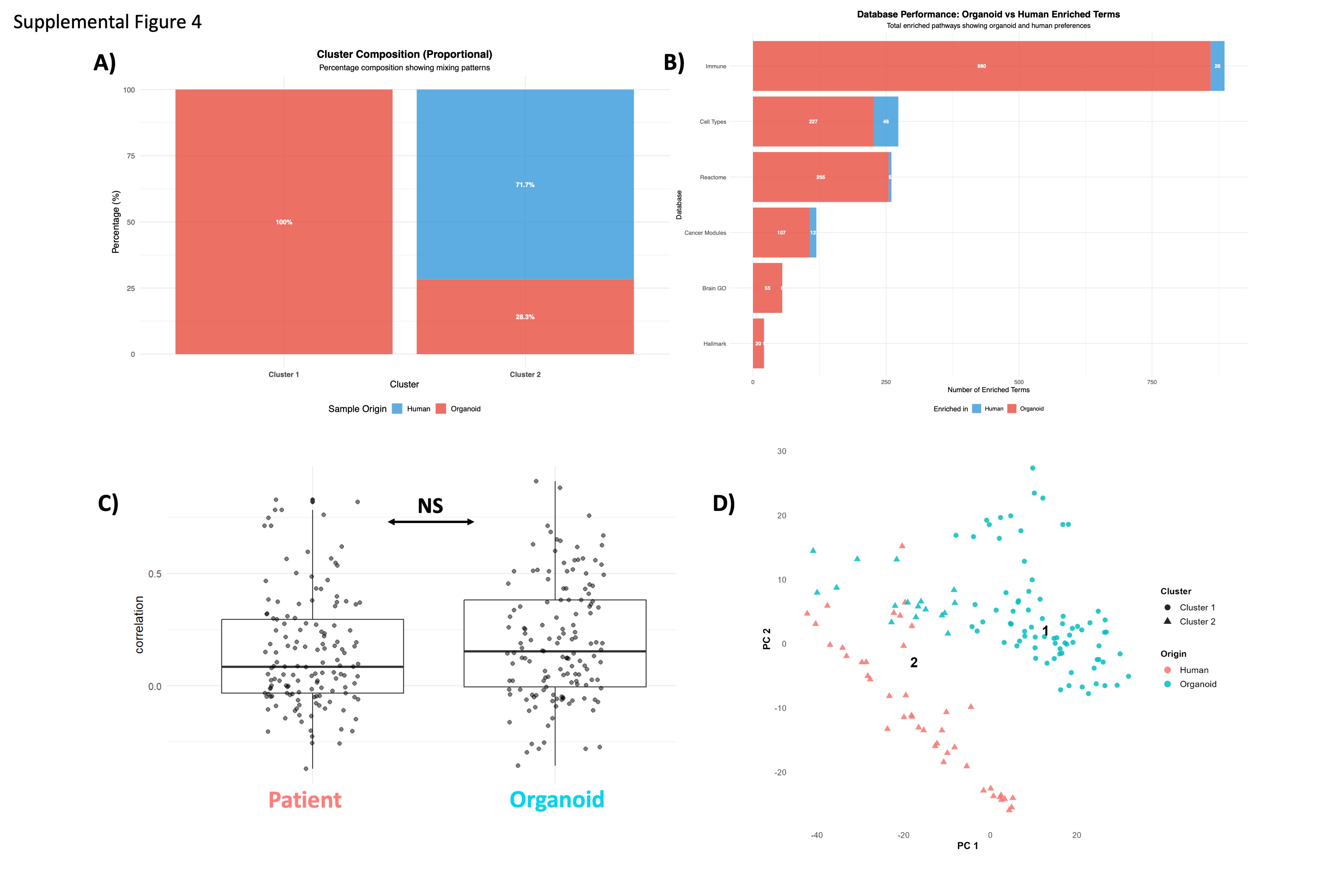

Supplement: Supplementary file 4 — Supplementary Material 4: Fig. 4: (A) bar chart showing percentage of ROIs within each cluster from either patient biopsies (human) or tumor seeded PNOs (PNO). (B) Enriched GSEA terms across databases (left) and by each experimental group (color). (C) Overall comparison between total correlation value of RNA to protein when all pairs are combined together between patient biopsy samples and tumor seeded PNOs. (D) PCA only based clustering of the patient biopsy sample vs. PNO using PC 1 and 2. [file 11060_2026_5515_MOESM4_ESM.jpeg]
